# Modeling Fabry nephropathy: insights from experimental systems towards precision nephrology

**DOI:** 10.1242/dmm.052716

**Published:** 2026-05-29

**Authors:** Hassan Elsaid, Jessica Furriol, Øystein Eikrem, Camilla Tøndel, Einar Svarstad

**Affiliations:** ^1^ Department of Clinical Medicine, University of Bergen, 5020 Bergen, Norway; ^2^ Department of Medicine, Haukeland University Hospital, 5021 Bergen, Norway; ^3^ Department of Pediatrics, Haukeland University Hospital, 5021 Bergen, Norway

**Keywords:** Fabry nephropathy, Experimental disease models, Kidney organoids, α-Galactosidase A deficiency, Precision nephrology

## Abstract

Fabry nephropathy is a major cause of kidney failure in Fabry disease, caused by pathogenic *GLA* variants that reduce α-galactosidase A activity and lead to globotriaosylceramide (Gb3) accumulation. In recent years, rapid advances in experimental modeling and emerging therapeutic strategies have dramatically reshaped understanding and treatment prospects for Fabry nephropathy. Experimental models – including cellular systems, patient-derived kidney organoids, and animal models such as zebrafish, *Drosophila*, mice and rats – have been pivotal in elucidating classic Gb3-dependent and novel Gb3-independent pathogenic mechanisms. These insights challenge the traditional Gb3-centric paradigm by highlighting other contributors, such as its metabolite lyso-Gb3, as well as α-synuclein accumulation and endothelial dysfunction. Despite established therapies for Fabry nephropathy, such as enzyme replacement and pharmacological chaperones, disease progression remains a clinical challenge, underscoring the urgent need for deeper mechanistic understanding and precision medicine approaches. This timely Review summarizes diverse model systems' unique strengths, limitations and translational potential to guide future research and therapeutic development for this complex disease.

## Teaser

**Summary:** Experimental models of Fabry nephropathy reveal complementary substrate-dependent and substrate-independent injury pathways, providing a translational framework to refine mechanism-driven therapies and advance precision nephrology.

## Introduction

**Fig. 1.**
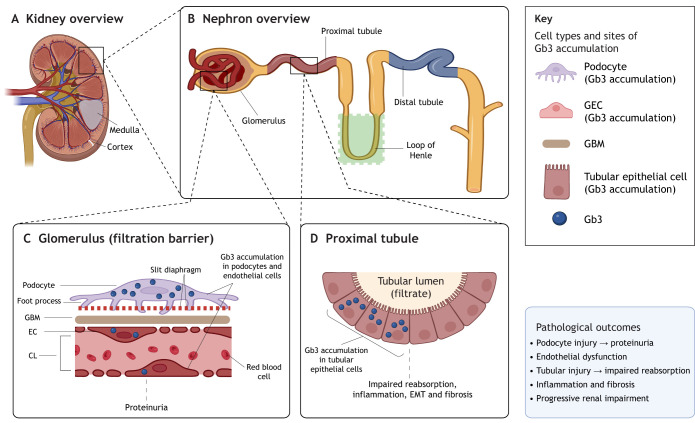
**Kidney structure and key renal cell types involved in Fabry nephropathy.** (A) Overview of kidney anatomy showing the cortex and medulla. (B) Simplified schematic of the nephron, the functional unit of the kidney, highlighting the glomerulus, proximal tubule, loop of Henle and distal tubule. (C) Enlarged view of the glomerular filtration barrier, including podocytes, the glomerular basement membrane and glomerular endothelial cells, which are major sites of Gb3 accumulation in Fabry nephropathy. Podocyte injury contributes to proteinuria. (D) Enlarged view of the proximal tubule, illustrating Gb3 accumulation in tubular epithelial cells and associated pathological consequences, including impaired reabsorption, inflammation, EMT and fibrosis. The figure highlights renal compartments and cell types most relevant to Fabry nephropathy, demonstrating that Gb3 accumulation across renal compartments contributes to cellular dysfunction and progressive kidney damage, and is intended to support accessibility for readers less familiar with kidney anatomy. Created in BioRender by Elsaid, H. (2026). https://BioRender.com/lb4mn72. This figure was sublicensed under CC-BY 4.0 terms. CL, capillary lumen; EC, endothelial cell; EMT, epithelial–mesenchymal transition; GBM, glomerular basement membrane; Gb3, globotriaosylceramide; GEC, glomerular endothelial cell.

Fabry disease is a progressive X-linked lysosomal storage disorder caused by deficient activity of the lysosomal enzyme α-galactosidase A, encoded by the galactosidase alpha (*GLA*) gene (Germain, 2000; Kok et al., 2021). In the kidney, the resulting lysosomal accumulation of glycosphingolipids, particularly globotriaosylceramide (Gb3), occurs in key renal cell types, including podocytes, glomerular endothelial cells and tubular epithelial cells, which are illustrated in the anatomical context of the nephron in Fig. 1 (Najafian et al., 2020; Ortiz et al., 2018; Tondel et al., 2015). This renal substrate accumulation disrupts cellular homeostasis and contributes to inflammation, fibrosis and cell death (Najafian et al., 2020; Rozenfeld et al., 2020; Tondel et al., 2015). Patients with Fabry nephropathy typically present with early-onset proteinuria and progressive glomerular and tubular dysfunction, often leading to end-stage renal disease if untreated (Kok et al., 2021; Najafian et al., 2011; Ortiz et al., 2018; Warnock and West, 2006).

**Fig. 2.**
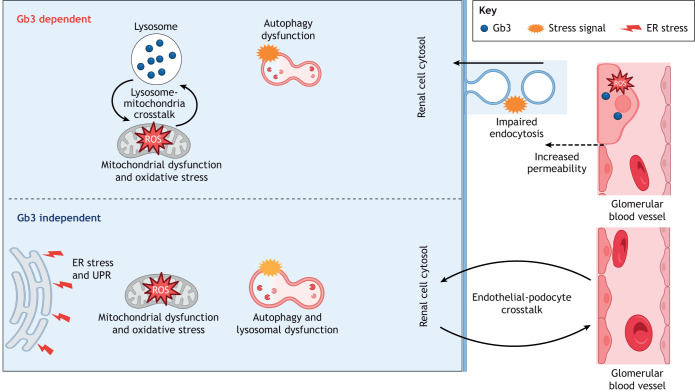
**Graphical summary of Gb3-dependent and partly Gb3-independent injury mechanisms in Fabry nephropathy.** The figure summarizes interconnected cellular injury pathways implicated in Fabry nephropathy. In the Gb3-dependent compartment, lysosomal Gb3 accumulation contributes to lysosome–mitochondria crosstalk, mitochondrial dysfunction and oxidative stress, autophagy dysfunction, impaired endocytosis and endothelial injury with increased permeability. In the Gb3-independent or partly substrate-independent compartment, α-galactosidase A deficiency and variant-specific protein misfolding can promote ER stress, UPR activation, mitochondrial oxidative stress, autophagy and lysosomal dysfunction, and pathological endothelial–podocyte crosstalk. These mechanisms should not be interpreted as fully separate pathways; rather, they may overlap, reinforce each other and vary according to model system, disease stage and *GLA* variant. Created in BioRender by Elsaid, H. (2026). https://BioRender.com/lb4mn72. This figure was sublicensed under CC-BY 4.0 terms. ER, endoplasmic reticulum; Gb3, globotriaosylceramide; ROS, reactive oxygen species; UPR, unfolded protein response.

Recent research has challenged the paradigm that Gb3 accumulation alone explains Fabry nephropathy. Experimental systems increasingly implicate mechanisms that may be partly Gb3 independent or downstream of substrate accumulation, including oxidative stress (Elsaid et al., 2023), impaired autophagy progression (Liebau et al., 2013), unfolded protein response (UPR) activation (Braunstein et al., 2020; Zivna et al., 2025) and α-synuclein toxicity (Braun et al., 2023). These interconnected Gb3-dependent and partly Gb3-independent injury pathways are summarized in Fig. 2 and provide a conceptual framework for interpreting the model-specific findings discussed below.

Despite the availability of disease-specific therapies, including enzyme replacement therapy (ERT) and pharmacological chaperones, clinical outcomes remain suboptimal, particularly in advanced disease stages, in which progression of renal dysfunction and persistent proteinuria are frequently observed (Germain et al., 2015; Rozenfeld et al., 2020; Tuttolomondo et al., 2021; Warnock et al., 2010). ERT involves intravenous administration of recombinant α-galactosidase A to reduce lysosomal substrate burden (Germain et al., 2015; Ortiz et al., 2018; Tondel et al., 2022), whereas pharmacological chaperones, such as migalastat, are small molecules that bind and stabilize specific misfolded α-galactosidase A variants in the endoplasmic reticulum (ER), allowing correct trafficking to lysosomes and partial restoration of residual enzyme activity in patients with amenable variants (Germain et al., 2012, 2019).

Experimental model systems – including genetically modified cell lines, patient-derived kidney organoids, and *in vivo* models ranging from *Drosophila* and zebrafish to rodents – have been central in dissecting disease mechanisms and evaluating therapeutic strategies in Fabry disease (Feriozzi and Rozenfeld, 2025; Rozenfeld et al., 2020). To provide a comprehensive and balanced overview, we surveyed the literature using PubMed and Web of Science databases, focusing on studies that model Fabry nephropathy across cellular, organoid and *in vivo* systems. Both mechanistic and translational studies were considered. Although previous reviews (Kok et al., 2021; Lenders and Brand, 2021; Tuttolomondo et al., 2021) have addressed Fabry disease pathogenesis and treatment, integrated analyses that systematically connect experimental model systems with mechanistic and translational insights remain relatively limited.

Here, we integrate insights across model systems to delineate key pathogenic mechanisms, assess their translational relevance and highlight future directions for model-informed precision nephrology.

## Cellular models of Fabry nephropathy

### Podocyte models

Podocytes are terminally differentiated epithelial cells that play a central role in maintaining the glomerular filtration barrier and are among the earliest and most severely affected cell types in Fabry nephropathy (Fall et al., 2016; Najafian et al., 2020; Tondel et al., 2008, 2015).

In a seminal study, Liebau et al. (2013) used RNA interference to suppress *GLA* expression in conditionally immortalized human podocytes, resulting in α-galactosidase A deficiency and intracellular Gb3 accumulation. This model demonstrated impaired autophagic flux, accompanied by downregulation of mechanistic target of rapamycin (mTOR) and AKT signaling pathways, indicating disruption of key cellular homeostasis mechanisms (Liebau et al., 2013). However, the precise molecular link between Gb3 accumulation and these downstream alterations remains incompletely understood. It has been proposed that lysosomal Gb3 overload may interfere with lysosome–mechanistic target of rapamycin complex 1 (mTORC1) signaling, alter membrane lipid composition affecting signaling platforms, or induce secondary mitochondrial and oxidative stress, thereby indirectly impairing autophagic processes (Aerts et al., 2008; Ballabio and Bonifacino, 2020; Elsaid et al., 2023; Rozenfeld et al., 2020). These possibilities highlight an important area for future investigation to determine whether such effects are direct consequences of substrate accumulation or arise from downstream cellular stress responses. Collectively, these findings support a link between lysosomal dysfunction and podocyte injury in Fabry nephropathy.

Complementary findings from human podocyte models of α-galactosidase A deficiency have further demonstrated that impaired autophagic flux contributes to mitochondrial dysfunction and amplifies cellular stress responses, reinforcing the link between lysosomal dysfunction and metabolic injury (Chung et al., 2021; Elsaid et al., 2023; Liebau et al., 2013).

Subsequent studies using similar small interfering RNA (siRNA)-based approaches in conditionally immortalized human podocyte cell lines reproduced these key features, including Gb3 accumulation and impaired autophagic flux (An et al., 2022; Kim et al., 2024). Importantly, intervention with polyethylene glycol-capped ceria–zirconia nanoparticles (PEG-CZNPs) restored autophagic flux, reduced oxidative stress, and attenuated apoptosis and profibrotic signaling (An et al., 2022; Kim et al., 2024). Mechanistically, PEG-CZNPs function as redox-active nanozymes capable of cycling between Ce^3+^ and Ce^4+^ states, enabling efficient scavenging of reactive oxygen species and restoration of intracellular redox balance (An et al., 2022; Kim et al., 2024). This reduction in oxidative stress is thought to alleviate secondary mitochondrial dysfunction and facilitate recovery of autophagic processes (Elsaid et al., 2023; Rozenfeld et al., 2020). In this context, PEG-CZNPs are primarily used as experimental tools to interrogate redox-dependent mechanisms of cellular injury, although their ability to reverse key pathological features highlights their potential as adjunctive therapeutic candidates targeting downstream stress pathways (An et al., 2022; Kim et al., 2024).

Gene-editing approaches have enabled the generation of more stable and experimentally tractable podocyte models. Pereira et al. (2016) developed *GLA*-deficient conditionally immortalized human podocyte cell lines (Saleem-derived) using clustered regularly interspaced short palindromic repeats/CRISPR-associated protein 9 (CRISPR/Cas9) gene editing. These cells exhibited widespread dysregulation of signaling pathways, including mitogen-activated protein kinase (MAPK), vascular endothelial growth factor (VEGF) and transforming growth factor beta (TGF-β) pathways, which are implicated in cellular stress responses, angiogenesis and fibrosis (Pereira et al., 2016; Rozenfeld et al., 2020). These models also demonstrated increased sensitivity to external stressors, supporting their utility as platforms for therapeutic screening (Pereira et al., 2016).

Similarly, Jehn et al. (2021) generated a stable CRISPR/Cas9-mediated *GLA*-knockout conditionally immortalized human podocyte cell line, resulting in near-complete loss of α-galactosidase A activity. These *GLA*-deficient cells displayed marked Gb3 accumulation and characteristic multilamellar inclusions within lysosomes, reflecting the typical storage phenotype observed in Fabry disease (Jehn et al., 2021). Proteomic analysis revealed persistent alterations in pathways related to lysosomal trafficking, metabolism and cell–cell interactions, even following ERT (Jehn et al., 2021). This suggests that α-galactosidase A deficiency induces broad and only partially reversible changes in podocyte biology, reinforcing the concept that disease progression is not solely dependent on substrate accumulation (Braun et al., 2019, 2023; Jehn et al., 2021).

These findings support earlier observations from human biopsy studies, which show that Gb3 accumulation in podocytes is associated with lysosomal enlargement and foot process effacement before overt glomerulosclerosis or clinical nephropathy, including in young patients with minimal albuminuria (Najafian et al., 2011, 2020; Tondel et al., 2008, 2015). The resulting structural alterations correlate with early-onset proteinuria and increased urinary podocyte loss, supporting an association between podocyte substrate accumulation, podocyte injury and detachment in Fabry nephropathy (Fall et al., 2016; Najafian et al., 2020).

Collectively, podocyte models indicate that Fabry nephropathy arises from a convergence of Gb3-dependent and Gb3-independent mechanisms (Braun et al., 2019, 2023; Liebau et al., 2013). While Gb3 accumulation contributes to lysosomal dysfunction and structural injury, downstream processes – including impaired autophagy, oxidative stress and α-synuclein-mediated proteotoxicity – may amplify or sustain disease progression, even in the setting of partial substrate clearance (Braun et al., 2019, 2023; Liebau et al., 2013). In particular, Braun et al. (2023) demonstrate that α-synuclein accumulation in CRISPR/Cas9-engineered *GLA*-deficient human podocyte cell lines localizes to lysosomal compartments and impairs lysosomal enzyme activity, disrupts autophagic flux and destabilizes cytoskeletal organization, thereby promoting cellular stress and podocyte detachment. Notably, inhibition of α-synuclein restored cellular function more effectively than Gb3 reduction alone, indicating that proteotoxic stress may act as an independent or self-sustaining driver of cellular injury (Braun et al., 2023). These findings suggest possible mechanistic overlap with other lysosomal storage and neurodegenerative disorders.

### Tubular and mesangial cell models

While podocytes represent a primary site of injury in Fabry nephropathy (Fall et al., 2016; Najafian et al., 2011, 2020), tubular epithelial and mesangial cells also play critical roles in disease progression, particularly in interstitial fibrosis and impaired protein reabsorption (Jeon et al., 2015; Rozenfeld et al., 2020, 2024). Cellular models targeting these compartments have provided key insights into how glycosphingolipid accumulation and downstream signaling pathways contribute to renal dysfunction beyond the glomerulus (Jeon et al., 2015; Labilloy et al., 2014; Thomaidis et al., 2009).

Labilloy et al. (2014) developed a polarized Madin-Darby canine kidney (MDCK) epithelial cell model, which resembles distal tubular/collecting duct epithelial cells, in which *GLA* expression was silenced using RNA interference. This resulted in intracellular Gb3 accumulation, lysosomal enlargement and disruption of lipid raft organization (Labilloy et al., 2014). These alterations impaired protein–protein interactions and membrane signaling, supporting the concept that Gb3 accumulation can interfere with epithelial polarity and endocytic trafficking in renal epithelial models (Labilloy et al., 2014). This provides a mechanistic basis for studying membrane-level dysfunction that may contribute to defective tubular handling in Fabry nephropathy.

In complementary models, Jeon et al. (2015) exposed human proximal tubular epithelial (HK-2) and mouse mesangial (SV40 MES13) cell lines to exogenously applied Gb3 and globotriaosylsphingosine (lyso-Gb3) (synthetic or purified analogs), inducing epithelial–mesenchymal transition (EMT), a process implicated in renal fibrosis. This response was mediated through activation of phosphoinositide 3-kinase (PI3K)/AKT and TGF-β signaling pathways, and pharmacological inhibition of TGF-β partially reversed these effects (Jeon et al., 2015). These findings highlight a link between glycosphingolipid exposure and profibrotic remodeling in renal cell models, supporting further investigation of anti-fibrotic strategies as potential adjuncts to substrate-directed therapies. Together with polarized epithelial models, these findings support a model in which Gb3 accumulation disrupts membrane organization and endocytic function, while Gb3 and lyso-Gb3 activate profibrotic signaling pathways that may contribute to interstitial fibrosis and disease progression (Jeon et al., 2015; Labilloy et al., 2014; Rozenfeld et al., 2020). Although lyso-Gb3 is a deacylated derivative of Gb3, its biological effects are best interpreted as downstream substrate-related signaling rather than strictly Gb3-independent mechanisms, as lyso-Gb3 can activate inflammatory, profibrotic and cellular stress pathways that may amplify and perpetuate injury beyond primary lysosomal substrate accumulation (Aerts et al., 2008; Jeon et al., 2015; Rozenfeld et al., 2020).

Fabry-related endothelial cell studies suggest that glycosphingolipid accumulation can increase the expression or release of pro-inflammatory and profibrotic mediators, including TGF-β1 and VEGF, which may contribute to renal cell injury and fibrotic remodeling (Lee et al., 2012; Rozenfeld et al., 2020). More broadly, glomerular endothelial cell and podocyte co-culture systems demonstrate that endothelial–podocyte crosstalk affects filtration barrier integrity and glomerular cell phenotype, highlighting a relevant axis for future Fabry nephropathy models (Hart et al., 2023).

Beyond substrate-driven toxicity, proteotoxic stress represents an additional mechanism of injury in Fabry disease. Consolato et al. (2022) demonstrated that expression of α-galactosidase A variants in *GLA*-deficient HEK293 cells – an epithelial-like human embryonic kidney cell line widely used for protein-processing studies – induces ER stress and activates the UPR, particularly via activating transcription factor 6 (ATF6) signaling in this Fabry model (Consolato et al., 2022). In this model, misfolded α-galactosidase A accumulates within the ER, triggering chronic stress signaling and impaired protein trafficking (Consolato et al., 2022). Notably, these effects were observed in the absence of significant Gb3 accumulation, indicating that certain pathogenic variants can drive cellular dysfunction through proteotoxic mechanisms independent of substantial substrate storage (Consolato et al., 2022). This highlights a variant-specific injury axis in Fabry disease, in which misfolding-induced stress may act in parallel with substrate-storage mechanisms.

Stable knockdown models in HK-2 cells further reinforce the contribution of chronic enzyme deficiency. Persistent α-galactosidase A depletion results in sustained intracellular Gb3 accumulation and reduced cell viability, with associated lysosomal storage abnormalities in renal epithelial cells (Schumann et al., 2021; Thomaidis et al., 2009). Importantly, Gb3 levels in renal tubular cells have been proposed as indicators of disease-related substrate burden and treatment response, supporting their potential use as candidate translational biomarkers (Selvarajah et al., 2011; Thomaidis et al., 2009).

Recent work has extended *GLA*-deficient HK-2 tubular epithelial models to therapeutic testing. In *GLA*-deficient HK-2 tubular epithelial cell lines, PEG-CZNPs restored autophagic flux and reduced oxidative stress, inflammation, apoptosis and profibrotic signaling, including TGF-β-associated pathways (An et al., 2022; Kim et al., 2024). These results are similar to those in the human podocyte cell line discussed above (An et al., 2022; Kim et al., 2024) and support the concept that targeting oxidative and autophagic dysfunction may complement substrate-directed therapies in preclinical Fabry models (An et al., 2022; Kim et al., 2024).

Collectively, tubular and mesangial cell models demonstrate that Fabry nephropathy extends beyond glomerular injury and involves substrate-associated epithelial membrane disruption and endocytic dysfunction (Labilloy et al., 2014), profibrotic signaling driven by Gb3 and lyso-Gb3 exposure (Jeon et al., 2015), proteotoxic stress induced by misfolded α-galactosidase A variants (Consolato et al., 2022), and oxidative and mitochondrial imbalance in renal epithelial models (An et al., 2022; Kim et al., 2024; Schumann et al., 2021). These systems provide mechanistic resolution at the cellular level while highlighting the need for more complex models to capture multicellular and systemic interactions.

## Advanced humanized systems: kidney organoids

Recent advances in stem cell biology have enabled the development of kidney organoids derived from human induced pluripotent stem cells (hiPSCs), providing an important bridge between reductionist cell models and *in vivo* systems (Morizane et al., 2015; Takasato et al., 2015). These three-dimensional systems recapitulate key aspects of human kidney development by self-organizing into nephron-like structures, including podocyte-like cells, proximal and distal tubular segments, and early glomerular-like compartments (Morizane et al., 2015; Takasato et al., 2015). They exhibit segment-specific marker expression, partial epithelial polarization and rudimentary filtration barrier features, thereby mimicking fundamental aspects of nephron organization and function (Morizane et al., 2015; Takasato et al., 2015; van den Berg et al., 2018). As such, they can offer a patient-specific platform to investigate disease heterogeneity, genotype–phenotype relationships and therapeutic responses (Cui et al., 2023a; Freedman et al., 2015; Kim et al., 2021). In Fabry nephropathy, kidney organoids have emerged as a valuable system for modeling cellular interactions and mechanisms that are difficult to capture in two-dimensional culture systems (Cui et al., 2023a; Kim et al., 2021).

### Gene-edited organoid models

Kim et al. (2021) generated kidney organoids from hiPSCs carrying CRISPR/Cas9-mediated disruption of *GLA*. These organoids recapitulated hallmark features of Fabry nephropathy, including Gb3 accumulation in podocyte-like and tubular compartments, increased oxidative stress, apoptosis and structural disorganization (Kim et al., 2021). Treatment with recombinant human α-galactosidase A reduced substrate accumulation and oxidative injury, while co-treatment with glutathione further improved organoid architecture and cellular survival, supporting a potential preclinical role for redox modulation as an adjunct to ERT (Kim et al., 2021).

These organoid-based observations provide important mechanistic insight into the role of oxidative stress in Fabry nephropathy. Increased reactive oxygen species production and depletion of antioxidant reserves, such as glutathione, were observed in *GLA*-mutant kidney organoids (Kim et al., 2021). At a cellular level, this redox imbalance may arise partly from mitochondrial dysfunction secondary to lysosomal impairment, leading to disrupted energy metabolism and further amplification of oxidative stress (Schumann et al., 2021). In turn, oxidative damage may impair autophagic flux and cellular repair mechanisms, creating a self-reinforcing cycle of injury (Liebau et al., 2013; Schumann et al., 2021). Although oxidative stress may initially arise downstream of Gb3 accumulation in organoid systems (Kim et al., 2021), findings from zebrafish models indicate that mitochondrial and oxidative injury can also occur in settings in which Gb3 accumulation is absent, supporting a dual role as both a consequence of substrate storage and a potential substrate-independent driver of disease progression (Elsaid et al., 2023).

### Patient-derived organoids

Cui et al. (2023a) extended this approach by generating kidney organoids from patient-derived hiPSCs carrying distinct *GLA* variants. These organoids reproduced genotype-dependent differences in disease severity, including variation in Gb3 accumulation, lysosomal abnormalities and oxidative injury, demonstrating the utility of organoids for modeling patient-specific disease heterogeneity (Cui et al., 2023a). Such models provide an important platform for precision approaches in Fabry nephropathy, particularly when variant-specific biology may influence treatment response.

In a complementary study, the same group used the same patient-derived kidney organoid platform and applied CRISPR/Cas9-mediated suppression of alpha 1,4-galactosyltransferase (P1PK blood group) (*A4GALT*), which encodes Gb3 synthase, to reduce substrate production directly (Cui et al., 2023b). This intervention significantly lowered Gb3 accumulation despite persistent α-galactosidase A deficiency, providing proof of concept for substrate reduction strategies that may complement ERT (Cui et al., 2023b). Importantly, these findings support a central role for Gb3 accumulation in driving key pathological features, reinforcing the relevance of Gb3-dependent mechanisms while also highlighting opportunities for therapeutic intervention upstream of lysosomal storage (Cui et al., 2023b).

### Clinical and research applications

Kidney organoids address several limitations of conventional two-dimensional cell systems by offering greater multicellular complexity, including podocyte-like cells, tubular compartments and endothelial precursors (Morizane et al., 2015; Takasato et al., 2015). In Fabry disease, patient-derived and gene-edited organoids enable modeling of *GLA* variant effects, substrate accumulation, oxidative injury and potentially individualized therapeutic responses (Cui et al., 2023a; Kim et al., 2021). These properties make organoids useful preclinical platforms for testing enzyme replacement strategies and antioxidants (Kim et al., 2021), substrate reduction approaches (Cui et al., 2023b) and, in principle, pharmacological chaperones or emerging gene-based therapies. Their ability to model genotype-dependent phenotypes further positions them as a potentially valuable platform for precision medicine and biomarker discovery (Cui et al., 2023a). More broadly, advances in kidney organoid technology have demonstrated the capacity of these systems to recapitulate key aspects of human nephrogenesis, including segment-specific nephron patterning and partial epithelial polarization (Low et al., 2019; Morizane et al., 2015; Takasato et al., 2015). Recent studies have further improved organoid fidelity through prolonged maturation and transplantation-based vascularization strategies (Tran et al., 2019; van den Berg et al., 2018), as well as microfluidic flow-based culture systems that enhance vascularization and maturation, thereby improving their physiological relevance for disease modeling beyond static early developmental organoids (Homan et al., 2019).

Beyond these applications, organoids provide an important intermediate platform between simple cell models and animal systems by enabling interrogation of multicellular responses and early tissue-level pathology in a human context (Freedman et al., 2015; Morizane et al., 2015; Takasato et al., 2015). This is particularly relevant in Fabry nephropathy, where disease progression likely reflects interactions among substrate accumulation, oxidative stress, altered signaling and chronic injury responses (Cui et al., 2023a; Kim et al., 2021; Rozenfeld et al., 2020). However, important limitations remain. Current kidney organoids often exhibit fetal-like immaturity (Takasato et al., 2015; Wu et al., 2018), incomplete vascularization (Low et al., 2019; van den Berg et al., 2018), and limited representation or maturation of certain nephron segments (Morizane et al., 2015; Wu et al., 2018), which may constrain their ability to model chronic disease progression. They also lack full immune, stromal and systemic inter-organ interactions, all of which are relevant to Fabry disease pathophysiology (Little and Combes, 2019; Rozenfeld et al., 2020). In addition, batch-to-batch variability and challenges in recapitulating long-term fibrotic remodeling remain significant barriers (Little and Combes, 2019; Wu et al., 2018).

Ongoing efforts to address these limitations – including co-culture with vascular components and transplantation-based vascularization (Low et al., 2019; van den Berg et al., 2018), improved extracellular matrix and engineered culture environments (Garreta et al., 2019), bioprinting technologies (Lawlor et al., 2021), and integration into organ-on-chip or flow-based systems (Homan et al., 2019) – may enhance the physiological relevance of future organoid systems. Until then, kidney organoids are best viewed as complementary rather than standalone models, with particular strength in bridging mechanistic discovery and translational therapeutic development.

## *In vivo* animal models

Animal models have been instrumental in elucidating the systemic consequences of α-galactosidase A deficiency, including renal and extrarenal manifestations in mice, zebrafish and rats (Elsaid et al., 2022; Miller et al., 2018; Ohshima et al., 1997). They have also supported preclinical evaluation of therapeutic interventions, including ERT, pharmacological chaperones and gene-based approaches (Ishii et al., 2004; Jung et al., 2001; Thurberg et al., 2002). The diversity of model organisms – from invertebrates to mammals – has enabled complementary interrogation of Fabry disease pathophysiology across evolutionary levels (Braunstein et al., 2020; Elsaid et al., 2022; Ohshima et al., 1997). Importantly, these systems capture multicellular, vascular and systemic interactions that cannot be fully reproduced *in vitro*, making them critical complementary tools for studying disease progression and translational therapeutic responses.

### *Drosophila melanogaster* (fruit fly)

*Drosophila melanogaster* offers powerful genetic tractability and rapid generation times, making it particularly well suited for mechanistic studies and drug screening. Braunstein et al. (2020) developed transgenic flies with systemic expression of pathogenic human α-galactosidase A variants A156V and A285D. In this model, the mutant α-galactosidase A proteins underwent ER retention, triggering ER stress, UPR activation and ER-associated degradation (Braunstein et al., 2020). Mechanistically, retention of misfolded enzymes within the ER is expected to disrupt protein-folding homeostasis and activate chronic UPR signaling, consistent with the canonical ER-stress response mediated through IRE1 (encoded by *ERN1*), PERK (encoded by *EIF2AK3*) and ATF6 pathways (Braunstein et al., 2020; Ron and Walter, 2007). Treatment with migalastat reduced ER stress/UPR activation and extended lifespan in this fly model (Braunstein et al., 2020). These findings highlight a Gb3-independent mechanism of injury driven by α-galactosidase A misfolding, ER retention and impaired proteostasis.

Although *Drosophila* lacks kidneys in the mammalian sense, *Drosophila* nephrocytes share important structural and functional similarities with human podocytes, including slit diaphragm-like filtration structures, endocytic activity, and roles in protein handling and cellular homeostasis (Helmstadter et al., 2017; Weavers et al., 2009). These similarities are particularly relevant given the central role of podocyte injury in Fabry nephropathy (Najafian et al., 2011, 2020). Although species-specific differences in glycosphingolipid metabolism and the absence of true nephron architecture limit direct disease modeling, nephrocyte systems provide valuable insight into conserved mechanisms of filtration-cell biology, lysosomal stress and proteotoxicity, supporting their use as complementary hypothesis-generating models (van de Leemput et al., 2022; Weavers et al., 2009).

Collectively, these findings highlight ER stress and UPR activation as contributors to cellular dysfunction in Fabry disease, particularly in the context of misfolded α-galactosidase A variants, and support a Gb3-independent, proteostasis-driven mechanism of injury (Braunstein et al., 2020; Consolato et al., 2022).

### *Danio rerio* (zebrafish)

Zebrafish provides a unique vertebrate model with conserved renal architecture, transparent embryos and suitability for high-throughput screening. Elsaid et al. (2022) generated zebrafish models with disrupted α-galactosidase A activity by targeting the zebrafish *gla* ortholog using CRISPR/Cas9-mediated gene editing. Notably, zebrafish lack Gb3 synthase activity and do not accumulate canonical Gb3, yet develop renal phenotypes including proteinuria, elevated creatinine and tubular pathology (Elsaid et al., 2022). Proteomic analysis of the *gla*^−/−^ zebrafish model further revealed mitochondrial dysfunction, altered energy metabolism and oxidative stress, supporting Gb3-independent renal injury mechanisms (Elsaid et al., 2023).

These findings provide strong evidence that Fabry-like renal injury can arise in the absence of canonical Gb3 accumulation, supporting the existence of Gb3-independent pathogenic mechanisms in this model (Elsaid et al., 2022, 2023). Rather than negating the importance of substrate accumulation, the zebrafish model suggests that α-galactosidase A deficiency disrupts broader sphingolipid homeostasis and cellular metabolism. In the absence of Gb3, alternative sphingolipid intermediates or altered lipid flux may perturb membrane composition, mitochondrial function and intracellular signaling pathways, contributing to oxidative stress and metabolic dysfunction; however, these mechanisms remain incompletely defined and require direct lipidomic validation (Elsaid et al., 2023). In parallel, proteostasis imbalance and lysosomal dysfunction may further amplify cellular injury independently of classical substrate storage, consistent with findings from human cell models showing persistent signaling, autophagy and proteostasis alterations despite reduced or absent substantial Gb3 accumulation (Braun et al., 2019; Consolato et al., 2022; Liebau et al., 2013).

These observations are supported by studies in systems in which Gb3 accumulation is absent, minimal, or experimentally reduced, but the evidence should be interpreted in the context of the model. In human podocyte models, enzyme replacement cleared Gb3 deposits but failed to normalize altered cellular signaling (Braun et al., 2019). In *GLA*-deficient HEK293 cells expressing pathogenic α-galactosidase A variants, ER stress and UPR activation occurred in the absence of significant Gb3 accumulation (Consolato et al., 2022). In zebrafish, loss of *gla* function produced mitochondrial and metabolic abnormalities despite the absence of canonical Gb3 accumulation (Elsaid et al., 2023). Together, these studies support the concept that Fabry nephropathy involves broader metabolic and proteostatic disturbances rather than Gb3 accumulation alone.

An additional distinguishing feature of the zebrafish model is the absence of a human *SNCA*/α-synuclein ortholog, despite the proposed role of α-synuclein accumulation in human Fabry podocyte pathology (Braun et al., 2023; Sun and Gitler, 2008). The persistence of renal phenotypes in zebrafish despite the absence of canonical Gb3 accumulation and a mammalian α-synuclein ortholog supports the existence of alternative, non-canonical mechanisms of injury (Elsaid et al., 2022, 2023). These may reflect fundamental consequences of α-galactosidase A deficiency on cellular metabolism and organelle function, rather than downstream effects of specific toxic substrates alone. As such, the zebrafish model provides an informative system for dissecting these pathways and generating hypotheses for therapeutic targets beyond substrate-directed approaches. Taken together, the zebrafish model provides strong *in vivo* evidence that Gb3 accumulation is not strictly required for the development of Fabry-like renal pathology.

### Murine models

Murine models have been among the most extensively used systems in Fabry disease research owing to ease of genetic manipulation, well-characterized renal physiology and compatibility with therapeutic testing (Ishii et al., 2004; Ohshima et al., 1997; Shiozuka et al., 2011). Although no single mouse model fully recapitulates human Fabry nephropathy, collectively, these models have provided critical insights into Gb3 substrate accumulation and lysosomal storage pathology (Ohshima et al., 1997; Shiozuka et al., 2011), renal compartment-specific injury (Maruyama et al., 2018; Taguchi et al., 2013), and therapeutic response to enzyme replacement, pharmacological chaperones and gene-based approaches (Ishii et al., 2004; Jung et al., 2001; Thurberg et al., 2002).

#### *Gla*-knockout mouse

Ohshima et al. (1997) developed the first Fabry mouse model through homologous recombination-mediated targeted disruption of the *Gla* gene, resulting in complete α-galactosidase A deficiency and progressive glycosphingolipid accumulation in multiple organs (Ohshima et al., 1997). These mice developed characteristic lamellar inclusion bodies (‘zebra bodies’), which are concentric, myelin-like lysosomal storage structures observed by electron microscopy and reflect intracellular glycosphingolipid accumulation (Ohshima et al., 1997; Thurberg et al., 2002). These inclusions were detected in renal cells, including glomerular podocytes and tubular epithelial cells, supporting the utility of this model for studying lysosomal storage pathology in Fabry disease (Ohshima et al., 1997; Thurberg et al., 2002). However, the renal phenotype of the original *Gla*-knockout mouse is relatively mild compared with that in human Fabry nephropathy, limiting its ability to model progressive glomerulosclerosis and advanced renal dysfunction.

Importantly, this model provided the foundation for early preclinical testing of ERT. Studies using recombinant α-galactosidase A demonstrated effective Gb3 clearance in vascular endothelial and some tubular compartments, but more limited clearance in podocytes, consistent with cell-type-specific differences in enzyme delivery or uptake (Christensen et al., 2007; Thurberg et al., 2002). These findings established both the therapeutic potential of ERT in reducing substrate burden and its limitations in reversing established renal storage pathology, particularly in cell types that are less accessible to circulating enzyme (Thurberg et al., 2002).

In addition to epithelial injury, Fabry mouse studies have highlighted vascular and endothelial contributions to renal pathology. In Fabry mouse kidney and cultured vascular endothelial cells, TGF-β1 and VEGF expression were increased and associated with apoptosis-related pathways, suggesting that endothelial-derived mediators contribute to Fabry nephropathy (Lee et al., 2012). More broadly, endothelial alterations are recognized contributors to renal fibrosis and microvascular injury, supporting further investigation of endothelial dysfunction in Fabry nephropathy models (Guerrot et al., 2012).

In addition to enzyme replacement strategies, this model has also been used to evaluate gene therapy approaches. Early studies using adeno-associated viral (AAV) vectors to restore wild-type *Gla* demonstrated sustained α-galactosidase A expression and reduction of glycosphingolipid accumulation across multiple organs, providing proof of concept for gene-based therapeutic strategies in Fabry disease (Jung et al., 2001).

#### Transgenic humanized mouse models

Ishii et al. (2004) generated transgenic mice expressing the human pathogenic α-galactosidase A variant R301Q on a *GLA*-null background, enabling the study of a misfolding-prone but catalytically competent variant *in vivo* (Ishii et al., 2004). Such misfolding-prone variants retain residual enzymatic activity but are destabilized, retained within the ER and inefficiently trafficked to lysosomes, making them potentially responsive to active-site-specific pharmacological chaperones (Fan and Ishii, 2007; Ishii, 2012). These models were, therefore, particularly valuable for evaluating migalastat (Ishii, 2012; Ishii et al., 2004). Treatment with migalastat increased α-galactosidase A activity and reduced Gb3 accumulation in disease-relevant tissues, including kidney and heart, in transgenic Fabry mouse models (Khanna et al., 2010). Importantly, these models demonstrated variant-dependent chaperone responsiveness, supporting the concept of precision treatment strategies in Fabry disease (Germain et al., 2012; Ishii et al., 2004). However, because these models are primarily biochemical rescue systems and do not reproduce robust nephropathy phenotypes, their utility lies mainly in target engagement, substrate reduction and biochemical validation rather than full pathophysiological modeling.

#### Gb3-synthase transgenic mouse

Shiozuka et al. (2011) generated transgenic mice overexpressing the human gene encoding Gb3 synthase (*A4GALT*), thereby increasing tissue Gb3 synthesis. This line was subsequently combined with α-galactosidase A-deficient backgrounds to generate high-substrate Fabry mouse models, including TgG3S^(+/−)^M^(+/−)^/KO and the symptomatic G3Stg/*GLA*ko model (Shiozuka et al., 2011; Taguchi et al., 2013). These models allow interrogation of the pathological consequences of excessive Gb3 burden in the setting of α-galactosidase A deficiency, rather than independently of enzyme deficiency. The G3Stg/*GLA*ko model displays markedly elevated Gb3 in major organs and renal tissue, providing a substrate-driven model of Fabry pathology (Shiozuka et al., 2011; Taguchi et al., 2013).

Follow-up studies using the same G3Stg/*GLA*ko model demonstrated progressive renal dysfunction, including albuminuria by 3 weeks, impaired urine concentrating ability at 5 weeks, polyuria by 10 weeks, and elevated blood urea nitrogen by 15 weeks (Taguchi et al., 2013). Histologically, these changes were accompanied by Gb3-filled lamellar inclusion bodies and marked vacuolation, predominantly within medullary thick ascending limb epithelial cells (Maruyama et al., 2018; Taguchi et al., 2013). Glomerular architecture remained largely preserved, supporting a predominantly tubular origin of injury in this model (Maruyama et al., 2018). Further mechanistic analyses revealed mitochondrial abnormalities, loss of apical membrane architecture and reduced expression of key transport proteins, including Na^+^/K^+^-ATPase, uromodulin and NKCC2, culminating in impaired salt and water reabsorption and peritubular fibrosis (Maruyama et al., 2018).

Collectively, these studies establish the G3Stg/*GLA*ko model as a useful system for investigating severe Gb3-driven tubular injury and progression of renal dysfunction (Maruyama et al., 2018; Taguchi et al., 2013). However, limited glomerular pathology and the possibility of overexpression-related artifacts underscore the need for caution when extrapolating directly to human Fabry nephropathy. Taken together, murine models remain important for mechanistic and therapeutic studies, particularly when interpreted as complementary systems with distinct strengths rather than as singular representations of human Fabry nephropathy.

#### *Gla*KO rat model

Miller et al. (2018) generated the first α-galactosidase A-deficient rat model using CRISPR/Cas9, providing a system that recapitulates several clinically relevant cardiorenal features of Fabry disease more closely than many conventional murine models (Miller et al., 2018). These rats exhibit progressive Gb3 and lyso-Gb3 accumulation, spontaneous tubular pathology, altered renal function and broader cardiorenal phenotypes, including cardiac valve abnormalities (Miller et al., 2018).

Compared with mouse models, the rat model provides improved representation of spontaneous renal disease progression and offers potential value for evaluating therapies targeting tubular injury and storage-driven mechanisms (Miller et al., 2018). As such, it serves as an important bridge between genetically tractable murine systems and more clinically representative disease modeling.

## Comparing complementary models of Fabry nephropathy

**Fig. 3.**
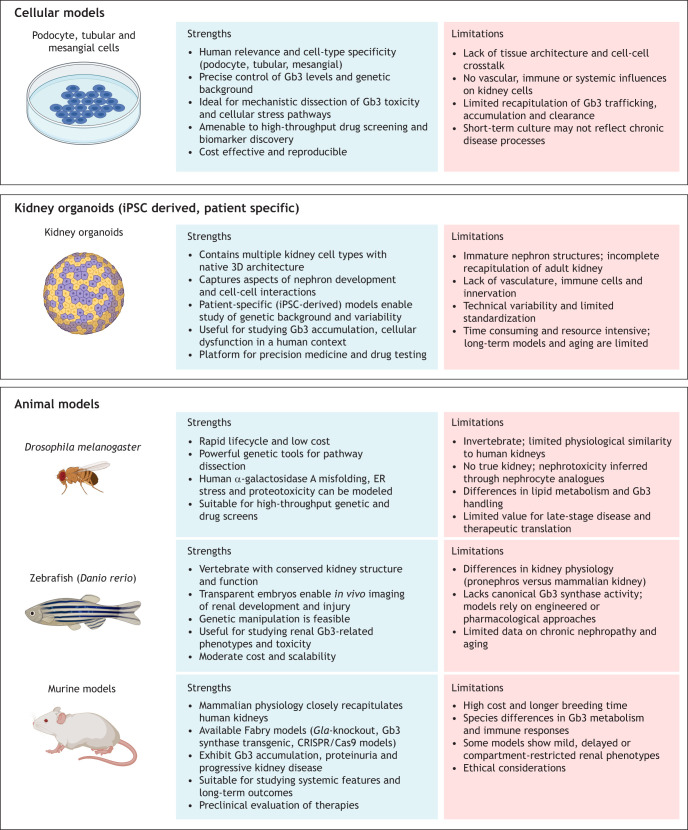
**Comparative strengths and limitations of experimental model systems used to study Fabry nephropathy.** The figure summarizes the complementary roles of cellular models, kidney organoids and animal models in Fabry nephropathy research. Cellular models, including podocyte, tubular and mesangial cell systems, provide high experimental control and are useful for mechanistic dissection and drug screening, but lack tissue architecture, vascular inputs, immune components and systemic physiology. Kidney organoids provide patient-specific, multicellular three-dimensional kidney-like systems, but remain limited by developmental immaturity, incomplete vascularization, technical variability and restricted modeling of long-term disease. Animal models provide *in vivo* context, with *Drosophila* supporting rapid genetic and proteotoxicity studies, zebrafish enabling vertebrate imaging and genetic manipulation, and murine models supporting mammalian physiology and preclinical therapeutic testing. No single model fully recapitulates Fabry nephropathy; therefore, integrated use of complementary systems is required to bridge molecular mechanisms and translational development. Created in BioRender by Elsaid, H. (2026). https://BioRender.com/lb4mn72. This figure was sublicensed under CC-BY 4.0 terms. CRISPR/Cas9, clustered regularly interspaced short palindromic repeats/CRISPR-associated protein 9; Gb3, globotriaosylceramide; ER, endoplasmic reticulum; iPSC, induced pluripotent stem cell.

Experimental models have been essential for elucidating how genetic and metabolic disturbances in Fabry disease translate into renal injury processes, providing critical insight into disease mechanisms and therapeutic targets (Elsaid et al., 2022; Kim et al., 2021; Liebau et al., 2013; Ohshima et al., 1997). The complementary strengths and limitations of these experimental systems across increasing levels of biological complexity are summarized in Fig. 3. No single model fully recapitulates the complexity of human Fabry nephropathy, and cellular, organoid and *in vivo* systems instead offer complementary perspectives, each capturing distinct dimensions of disease biology (Fig. 3, Table 1). Table 1 provides a mechanism-oriented comparison of these systems, highlighting the relative contribution and temporal emergence of Gb3-dependent and Gb3-independent pathways. Understanding their relative strengths and constraints is, therefore, critical for interpreting experimental findings and guiding future model development.

**Table 1.** Mechanism-oriented comparison of experimental models of Fabry nephropathy

Model system	Gb3 accumulation	Gb3-dependent mechanisms	Gb3-independent mechanisms	Relative mechanism prominence*	Temporal onset of pathology^‡^	Translational relevance	References
Podocyte cell lines (human, *GLA* deficient)	Low–moderate (variable)	Lysosomal dysfunction, autophagy impairment	α-Synuclein accumulation, proteotoxic stress, mitochondrial dysfunction	Mixed; Gb3-independent mechanisms prominent in selected models	Early cellular stress; timing varies by model	High (human-derived, mechanistic resolution)	Braun et al., 2023; Jehn et al., 2021; Liebau et al., 2013; Pereira et al., 2016
Tubular epithelial/mesangial cell lines (HK-2, SV40 MES13)	Moderate (exogenous or induced)	Lipid accumulation, membrane disruption and profibrotic signaling	ER stress, UPR activation, oxidative stress	Mixed (context dependent)	Early–intermediate	Moderate (reductionist systems)	An et al., 2022; Jeon et al., 2015; Kim et al., 2024; Labilloy et al., 2014; Selvarajah et al., 2011; Thomaidis et al., 2009
Kidney organoids (hiPSC derived)	Moderate–high	Lysosomal storage, structural nephron defects	Oxidative stress, mitochondrial dysfunction	Mixed	Intermediate	High (human, multicellular)	Cui et al., 2023a,b; Kim et al., 2021
*Drosophila* nephrocyte model	Low–moderate	Limited direct Gb3 storage	Proteostasis imbalance, ER stress, cytoskeletal dysfunction	Gb3-independent dominant	Early	Moderate (functional analog model)	Braunstein et al., 2020
Zebrafish (*gla*^−/−^)	Absent	Minimal	Altered sphingolipid metabolism, mitochondrial dysfunction and proteostasis imbalance	Gb3-independent dominant	Early (developmental stage)	High (*in vivo*, whole organism)	Elsaid et al., 2022; Elsaid et al., 2023
*Gla*-knockout mouse	High	Lysosomal storage, endothelial and tubular dysfunction	Secondary oxidative stress, inflammation	Gb3-dependent dominant	Intermediate–late	Very high (preclinical standard)	Ohshima et al., 1997
Transgenic/humanized mouse models	High (variant dependent)	Substrate accumulation (variant specific)	Proteotoxic stress (misfolded variants), ER retention	Mixed (variant dependent)	Variable	Very high (precision modeling)	Ishii et al., 2004
Gb3 synthase overexpression mouse (G3Stg/*GLA*ko)	Very high (driven by increased synthesis)	Tubular Gb3 overload, lysosomal dysfunction, epithelial injury, fibrosis	Secondary mitochondrial dysfunction, metabolic stress	Gb3-dependent dominant	Early–intermediate (rapid tubular phenotype)	High (models severe substrate-driven pathology)	Maruyama et al., 2018; Shiozuka et al., 2011; Taguchi et al., 2013
*Gla*KO rat (Dark Agouti)	High (Gb3 and lyso-Gb3)	Lysosomal storage, tubular injury, cardiorenal dysfunction	Oxidative stress, metabolic dysfunction (likely secondary)	Gb3-dependent dominant (with secondary mechanisms)	Intermediate–late (progressive phenotype)	Very high (closest to human renal pathology among rodent models)	Miller et al., 2018

*Relative mechanism prominence reflects the dominant pathological drivers observed in each model system based on current evidence. ^‡^Temporal onset refers to the stage at which detectable pathological features emerge relative to model development or intervention. ER, endoplasmic reticulum; Gb3, globotriaosylceramide; *Gla*KO, *Gla* knockout; hiPSC, human induced pluripotent stem cell; lyso-Gb3, globotriaosylsphingosine; SRT, substrate reduction therapy; UPR, unfolded protein response.

Two-dimensional cellular models have been particularly valuable for dissecting defined molecular mechanisms, including substrate-associated epithelial membrane disruption (Labilloy et al., 2014), oxidative and autophagic dysfunction (An et al., 2022; Liebau et al., 2013), proteotoxic stress and UPR activation (Consolato et al., 2022), and profibrotic signaling induced by Gb3 and lyso-Gb3 exposure (Jeon et al., 2015). Their strengths lie in experimental control, precise genetic manipulation, and suitability for high-resolution mechanistic and therapeutic studies (Jehn et al., 2021; Pereira et al., 2016). However, their reductionist nature limits their ability to capture multicellular interactions, vascular contributions and spatial organization of renal tissue. In particular, endothelial–podocyte or endothelial–epithelial crosstalk, which has been implicated in Fabry-associated renal injury, remains incompletely represented in most two-dimensional systems (Hart et al., 2023; Lee et al., 2012). Future advances incorporating multicellular co-culture systems, microphysiological platforms and kidney-on-a-chip technologies may help bridge this gap while preserving mechanistic precision.

Kidney organoids represent an important intermediate platform, introducing greater biological complexity, patient-specific modeling and multicellular organization not achievable in conventional cell systems (Morizane et al., 2015; Takasato et al., 2015). Their ability to recapitulate aspects of nephron patterning and genotype-dependent Fabry phenotypes makes them valuable for precision-medicine-oriented disease modeling (Cui et al., 2023a; Kim et al., 2021). However, limitations related to developmental immaturity, incomplete vascularization and restricted modeling of chronic fibrotic progression remain significant challenges (Little and Combes, 2019; Wu et al., 2018). Notably, endothelial and immune components are still incompletely represented, limiting the ability to model vascular dysfunction and inflammatory responses relevant to Fabry nephropathy (Little and Combes, 2019; Rozenfeld et al., 2020). Continued development of vascularized organoids, engineered extracellular matrices and microfluidic integration may enhance physiological relevance and long-term disease modeling (Garreta et al., 2019; Homan et al., 2019; van den Berg et al., 2018).

Non-mammalian *in vivo* systems, including *Drosophila* and zebrafish, provide complementary advantages for interrogating conserved cellular mechanisms and uncovering non-canonical injury pathways. *Drosophila* models have been informative for studying α-galactosidase A misfolding, ER stress and proteotoxicity (Braunstein et al., 2020), whereas zebrafish models have provided evidence for mitochondrial and metabolic injury in the absence of canonical Gb3 accumulation (Elsaid et al., 2022, 2023). However, species-specific differences – including the absence of mammalian nephron architecture in *Drosophila*, the absence of canonical Gb3 synthesis in zebrafish, and broader differences in sphingolipid metabolism – limit their ability to fully model progressive human kidney disease (Elsaid et al., 2023; Weavers et al., 2009). As such, their greatest value lies in hypothesis generation and mechanistic discovery, which can then be validated in more physiologically representative systems.

Mammalian models, including murine and rat systems, provide the closest approximation to human renal physiology among current *in vivo* Fabry models and remain important for studying multicellular injury, systemic interactions and therapeutic responses (Miller et al., 2018; Ohshima et al., 1997). These models have been instrumental in evaluating ERT (Christensen et al., 2007; Thurberg et al., 2002), pharmacological chaperones (Ishii et al., 2004; Khanna et al., 2010) and gene-based approaches (Jung et al., 2001). They have also highlighted vascular and endothelial contributions to renal pathology, including increased TGF-β1 and VEGF signaling in Fabry mouse kidney and endothelial-cell contexts (Lee et al., 2012). However, species-specific differences in disease severity, renal compartment involvement, immune responses and temporal progression can limit direct translation to human disease (Maruyama et al., 2018; Miller et al., 2018; Ohshima et al., 1997; Taguchi et al., 2013). Future refinement of these models – including improved representation of glomerular pathology, incorporation of genetic diversity and modeling of sex-dependent and longitudinal disease trajectories – will be important to enhance predictive value.

Taken together, current experimental systems are best viewed not as isolated models but as components of an integrated framework. Although each system has inherent constraints, their combined use enables a more comprehensive understanding of Fabry nephropathy across molecular, cellular and organismal scales. Future progress will depend on developing physiologically faithful, scalable and precision medicine-compatible models. Advances in induced pluripotent stem cell (iPSC)-derived organoids – including improved vascularization and maturation – together with microfluidic kidney-on-a-chip platforms that better model perfusion and epithelial polarization offer promising avenues to enhance physiological relevance (Homan et al., 2019; Low et al., 2019; van den Berg et al., 2018). In parallel, CRISPR/Cas9-based engineering enables precise modeling of patient-specific *GLA* variants and substrate-reduction strategies in kidney organoids, facilitating personalized disease modeling and therapeutic screening (Cui et al., 2023a,b; Kim et al., 2021). Leveraging these complementary innovations will be essential for improving translational accuracy and advancing precision nephrology in Fabry disease.

## Translational implications and future directions in Fabry nephropathy

**Fig. 4.**
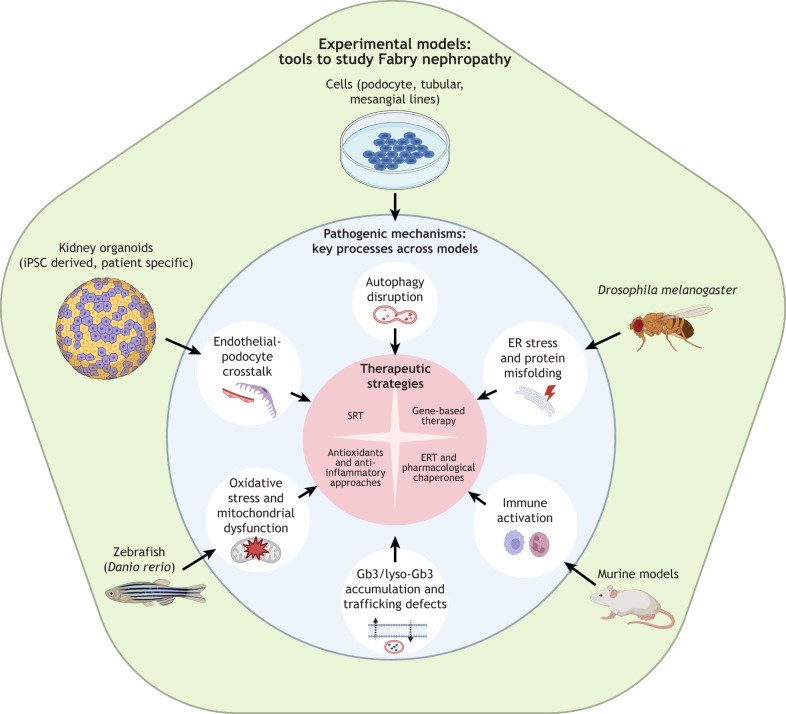
**Integrated framework linking experimental models, pathogenic mechanisms and therapeutic strategies in Fabry nephropathy.** Experimental model systems, including cellular models, kidney organoids, *Drosophila melanogaster*, zebrafish and murine models, provide complementary platforms for identifying pathogenic mechanisms and evaluating therapeutic strategies in Fabry nephropathy. The outer ring summarizes the model systems used to study Fabry nephropathy. The middle ring highlights major pathogenic processes identified across these systems, including autophagy disruption, ER stress and protein misfolding, oxidative stress and mitochondrial dysfunction, inflammation and immune activation, endothelial–podocyte crosstalk, and Gb3/lyso-Gb3 accumulation and trafficking defects. The central core summarizes therapeutic strategies informed by these mechanisms, including ERT and pharmacological chaperones, SRT, gene-based therapies, and antioxidant or anti-inflammatory approaches. The figure emphasizes that model systems, mechanisms and therapeutic strategies should be interpreted as interconnected rather than linear or isolated categories. Created in BioRender by Elsaid, H. (2026). https://BioRender.com/lb4mn72. This figure was sublicensed under CC-BY 4.0 terms. ER, endoplasmic reticulum; ERT, enzyme replacement therapy; Gb3, globotriaosylceramide; iPSC, induced pluripotent stems cell; lyso-Gb3, globotriaosylsphingosine; SRT, substrate reduction therapy.

Experimental model systems have expanded the therapeutic framework for Fabry nephropathy by linking molecular mechanisms to candidate therapeutic targets. This model-to-mechanism-to-therapy framework is summarized in Fig. 4. Rather than supporting a single-pathway model driven solely by Gb3 accumulation, these experimental systems collectively support the view that Fabry nephropathy involves interacting processes, including lysosomal dysfunction and impaired autophagy (Liebau et al., 2013), oxidative and mitochondrial stress (Elsaid et al., 2023; Kim et al., 2021; Schumann et al., 2021), and proteotoxic stress linked to misfolded α-galactosidase A variants (Braun et al., 2023; Braunstein et al., 2020; Consolato et al., 2022). This conceptual shift has supported the diversification of therapeutic strategies beyond conventional enzyme replacement.

ERT, including agalsidase-α and agalsidase-β, remains a clinical cornerstone for Fabry disease management (Germain et al., 2015; Ortiz et al., 2018). As described in murine model systems, recombinant α-galactosidase A can clear Gb3 from vascular endothelial and some tubular compartments, but clearance is more limited in podocytes and varies by renal cell type (Christensen et al., 2007; Thurberg et al., 2002). This restricted penetrance is consistent with cell-type-specific differences in enzyme distribution and uptake within the kidney (Christensen et al., 2007; Lenders and Brand, 2021). In kidney organoids, recombinant human α-galactosidase A reduced Gb3 accumulation and oxidative stress, although additional glutathione treatment further improved organoid architecture and cell survival, indicating that substrate clearance alone may not fully resolve downstream stress pathways (Kim et al., 2021). These findings help explain why proteinuria and renal dysfunction may persist in some patients despite ERT, particularly in advanced disease stages (Germain et al., 2015; Warnock et al., 2010).

A clinically important advance in this context is pegunigalsidase alfa, a PEGylated, plant cell-expressed recombinant α-galactosidase A approved for treatment of adults with Fabry disease (European Medicines Agency product information; U.S. Food and Drug Administration prescribing information). Preclinical and early clinical studies indicate that pegunigalsidase alfa has an extended plasma half-life and altered pharmacokinetic profile compared with earlier ERT formulations (Schiffmann et al., 2019). However, direct evaluation in advanced kidney-specific experimental systems, such as Fabry kidney organoids, remains limited. Therefore, potential advantages for difficult-to-reach renal compartments, including podocytes, should be considered a hypothesis requiring direct validation in human-relevant models.

Pharmacological chaperones, particularly migalastat, represent a precision-medicine approach for patients with amenable *GLA* variants. Studies in HEK293 cells and *Drosophila* models demonstrate that chaperone-mediated stabilization of misfolded α-galactosidase A can reduce ER stress and improve enzyme folding or activity (Braunstein et al., 2020; Consolato et al., 2022). In transgenic TgM/KO mice, migalastat increased α-galactosidase A and reduced Gb3 accumulation in disease-relevant tissues, including kidney and heart (Ishii et al., 2004; Khanna et al., 2010). These findings support proteostasis modulation as a therapeutic axis and emphasize the importance of variant-specific amenability testing for genotype-guided treatment selection (Kobayashi, 2024; Lukas et al., 2020).

Substrate reduction therapy (SRT) offers a complementary strategy by limiting glycosphingolipid synthesis rather than replacing α-galactosidase A activity (van der Veen et al., 2020). In Fabry kidney organoids, CRISPR/Cas9-mediated suppression of *A4GALT*, which encodes Gb3 synthase, significantly reduced Gb3 accumulation despite persistent enzyme deficiency, providing proof of concept for substrate reduction in a human organoid model (Cui et al., 2023b). Conversely, Gb3 synthase transgenic mouse models demonstrate that increased Gb3 synthesis on an α-galactosidase A-deficient background can drive severe substrate-loaded renal pathology, particularly tubular injury (Maruyama et al., 2018; Shiozuka et al., 2011; Taguchi et al., 2013). Together, these findings support SRT as a rational adjunct to ERT when substrate burden remains inadequately controlled.

Beyond substrate-centric approaches, experimental models have uncovered additional therapeutic vulnerabilities. Oxidative stress and mitochondrial dysfunction are observed in Fabry kidney organoids and renal epithelial models (Kim et al., 2021; Schumann et al., 2021). In *GLA*-mutant kidney organoids, glutathione co-treatment improved organoid architecture and cell survival beyond recombinant α-galactosidase A treatment alone, supporting redox modulation as a potential adjunctive strategy (Kim et al., 2021). In Fabry-relevant cellular and animal models, the redox-active nanozymes, PEG-CZNPs, reduced oxidative stress, inflammation, apoptosis and TGF-β-associated profibrotic signaling, while restoring autophagic flux and improving cell viability (An et al., 2022; Kim et al., 2024). These findings position nano-antioxidant strategies as promising preclinical adjuncts to substrate-targeting therapies.

Proteotoxic stress represents an additional therapeutic axis. Misfolded α-galactosidase A variants can induce ER stress and UPR activation in Fabry-relevant cell and fly models (Braunstein et al., 2020; Consolato et al., 2022; Zivna et al., 2025). Notably, α-synuclein accumulation has emerged as a mediator of podocyte injury, with α-synuclein inhibition restoring cellular function more effectively than substrate reduction alone in a Fabry podocyte model (Braun et al., 2023). These observations support further evaluation of combination strategies that address both primary enzymatic deficiency and downstream cellular stress responses.

Gene therapy represents a potentially transformative approach, aiming to achieve sustained endogenous enzyme production. In *Gla*-deficient murine models, AAV delivery of wild-type *Gla* achieved long-term α-galactosidase A expression and reduced glycosphingolipid accumulation across multiple organs (Jung et al., 2001). Early-phase clinical studies using lentiviral-modified autologous hematopoietic stem cells have reported sustained enzyme activity and partial renal substrate clearance in patients with Fabry disease (Khan et al., 2021). Although kidney organoids and humanized mouse models are promising platforms for evaluating vector tropism, transgene durability and off-target effects, their application to Fabry-specific gene therapy testing remains an area for further development.

Accumulating evidence indicates that different *GLA* variant classes may drive disease through distinct but overlapping mechanisms. Misfolding-prone variants can promote ER retention, UPR activation and impaired proteostasis (Consolato et al., 2022; Ishii et al., 2004; Zivna et al., 2025), whereas variants causing severe loss of α-galactosidase A activity more directly promote substrate accumulation and lysosomal dysfunction (Ohshima et al., 1997; Ortiz et al., 2018). This mechanistic heterogeneity highlights the importance of variant-specific therapeutic strategies and empirical amenability testing in Fabry disease (Kobayashi, 2024; Lukas et al., 2020).

Despite these advances, experimental systems also highlight critical translational gaps. Most models incompletely capture long-term disease progression, immune responses and intercellular signaling dynamics that drive fibrosis and irreversible kidney damage. In particular, endothelial–podocyte crosstalk and inflammatory amplification loops remain underrepresented despite their likely contribution to disease progression (Hart et al., 2023; Lee et al., 2012; Rozenfeld et al., 2020). Emerging systems such as vascularized organoids and microfluidic kidney-on-a-chip platforms offer opportunities to address these limitations by integrating hemodynamic forces and multicellular interactions (Homan et al., 2019; Low et al., 2019; van den Berg et al., 2018).

Looking forward, the field should move from single-modality interventions toward mechanism-informed, multi-target therapeutic strategies. Combining substrate clearance with modulation of oxidative stress, proteostasis and inflammatory signaling may be required to achieve durable clinical benefit in patients with established renal injury. Equally important is the development of precision model systems, including patient-specific organoids and gene-edited platforms, to stratify therapeutic responses and guide individualized treatment decisions (Cui et al., 2023a,b; Kim et al., 2021).

In summary, experimental models have moved the field beyond a reductionist view of Fabry nephropathy as solely a consequence of substrate accumulation toward a systems-level understanding of disease. Fabry nephropathy is increasingly understood as a complex, multi-layered disorder in which lysosomal dysfunction, metabolic imbalance, proteostasis disruption and maladaptive intercellular communication collectively contribute to renal injury. The next phase will depend on integrating this biological complexity with translational scalability to bridge the gap between experimental insight and clinical impact.
